# Ambulatory Heart Rate Variability Monitoring in Depressed and Suicidal Adolescents Receiving Intensive Outpatient Treatment: Feasibility Study

**DOI:** 10.2196/99624

**Published:** 2026-08-07

**Authors:** Lori N Scott, Tina R Goldstein, Lillian L Manna, Dawn Rice, Olaoluwa Owoputi, Peter L Franzen

**Affiliations:** 1 Department of Psychiatry, School of Medicine, University of Pittsburgh, 3811 O'Hara Street, Pittsburgh, PA, 15213, United States, 1 4126484565; 2 University of Pittsburgh Medical Center, Pittsburgh, PA, United States

**Keywords:** heart rate variability, ambulatory assessment, adolescents, wearable sensors, feasibility, suicidal ideation, depression

## Abstract

Continuous ambulatory HF-HRV (high-frequency heart rate variability) monitoring using a chest-worn ECG (electrocardiogram) device met a priori feasibility benchmarks (≥75% compliance and data quality) in adolescents at high risk for suicide over approximately two to three weeks, including during school days and sleep.

## Introduction

Suicide risk in adolescence fluctuates over short time scales and is shaped by affective and contextual processes [1]. Continuous naturalistic assessment of psychophysiological markers may improve understanding of proximal risk states and inform timely intervention strategies [2]. High-frequency heart rate variability (HF-HRV; 0.15‐0.40 Hz), reflecting parasympathetic nervous system activity, has been linked to emotion regulation capacity and affective flexibility [3]. Lower resting HF-HRV and altered HF-HRV reactivity have been associated with suicidal ideation and behavior beyond depressive and anxiety symptoms [4-6].

However, most studies linking HF-HRV to suicide risk have relied on laboratory or cross-sectional designs. Ambulatory monitoring may better capture real-world fluctuations in suicidal ideation, which often emerge outside clinical settings and may be influenced by sleep and daily demands [1,7]. Chest-worn electrocardiogram (ECG) sensors provide superior signal quality compared with wrist-based photoplethysmography devices [8], yet no prior studies with adolescents used chest-worn devices for more than one week. Chest-worn devices may pose feasibility challenges including discomfort, visibility under clothing, interference with daily activities, and susceptibility to user error, especially among distressed adolescents. Hence, the long-term feasibility for their use in adolescents receiving intensive psychiatric care remains unclear.

This study evaluated the feasibility of continuous HF-HRV monitoring over 2‐3 weeks using a research-grade chest-worn ECG sensor in adolescents receiving intensive outpatient treatment for depression and suicidality. Our target benchmarks, specified a priori, were ≥75% for both compliance (sensor wear time) and data quality (valid HRV data). We also examined whether feasibility varied over time, by sleep and wake periods, or by school attendance.

## Methods

### Participants and Procedures

Participants were 121 adolescents (ages 13‐18) enrolled between September 2021 and October 2023 from intensive outpatient and partial hospitalization programs specializing in depression and suicidality. Eligibility required endorsement of suicidal thoughts in the past month per self-report or clinical evaluation.

Following baseline questionnaires and clinical interviews, participants were asked to wear a chest-worn ECG sensor (Movisens ECGMove4; 1024 Hz) on an elastic strap continuously for 14 days, with the option to continue for an additional 7 days. As an alternative, 6% of participants elected to use disposable chest electrodes. Participants were instructed to charge the ECG sensor daily (~10‐15 min) and to remove it for activities that risked damage. Participants received escalating daily compensation ($5‐10/d) contingent on ≥20 hours of wear. ECG data were analyzed in 60-second epochs using automated R-peak detection and artifact handling in DataAnalyzer software (Movisens GmbH, Germany). The software also provided automatic detection of sensor wear (per epoch) based on acceleration data. Compliance was defined as the proportion of intended monitoring minutes during the scheduled monitoring period during which the sensor was worn. Data quality was defined as the proportion of valid HRV values produced by the DataAnalyzer software during periods when the sensor was worn.

Participants also wore research-grade wrist actigraphs (CentrePoint Insight Watch, Ametris, Pensacola FL) to collect daily sleep data. Sleep periods (including naps) were identified using standard algorithms (Cole-Kripke and Tudor-Locke) in ActiLife software version 6.13.5 (Ametris, Pensacola, FL). Actigraphy data were available for 97.7% of ECG measurement minutes (of which 35.2% were during sleep).

Participants also completed morning and evening daily diary surveys via SMS or email links to a secure online platform (n=1485 days [91.8%] completion). Evening diary items included school attendance using predefined categories (in-person, virtual, no school, nonschool day).

### Data Analysis

Descriptive statistics summarized feasibility outcomes. Bivariate correlations, *χ*^2^ tests, and *t*-tests explored demographic and clinical differences in feasibility metrics. Linear mixed-effects models (restricted maximum likelihood; SPSS version 29.0) examined time (days), sleep vs wake, and school attendance effects on compliance and data quality.

### Ethical Considerations

This study was approved by the University of Pittsburgh Institutional Review Board (STUDY20110313). Adolescents provided assent, and parents provided written informed consent.

## Results

Demographic and clinical characteristics are shown in Table 1. Of 121 enrolled adolescents, 100 (82.6%) provided ECG data. Among those without ECG data, 9 declined due to discomfort and 12 agreed but never wore the sensor. Participants without ECG data had marginally lower SES (*t*(119) = 1.96, *P*=.05) and were more likely to identify as non-White (*χ^2^*(1)=5.22, *P*=.02); they did not differ on age, sex at birth, ethnicity, age at depression onset, level of suicidal ideation, or suicide attempt history (see Table 1).

**Table 1.** Demographic and clinical characteristics. Socioeconomic status was assessed using the Hollingshead Four-Factor Index of Social Status [9]. Percentages may not sum to 100% due to missing data. All participants endorsed suicidal thoughts at study enrollment per the participant's treatment team or self-report in the past month and were allowed to continue in the study even if they denied suicidal ideation in the C-SSRS^a^ interview.

Characteristics	Participants (n=121)		Missing ECG^d^ (n=21)		Not missing ECG (n=100)		*P* value
	Mean/n	SD/%	Mean/n	SD/%	Mean/n	SD/%	
Age at consent (mean)	15.86	1.51	16.11	1.68	15.81	1.48	.40
Sex at Birth							.39
Male	26	21.5	6	28.6	20	20.0	
Female	95	78.5	15	71.4	80	80.0	
Ethnicity							
Non-Hispanic/LatinX	114	94.2	21	100.0	93	93.0	.21
Hispanic/LatinX	7	5.8	0	0.0	7	7.0	
Race							.02^b^
Asian	4	3.3	1	4.8	3	3.0	
Black	5	4.1	3	14.3	2	2.0	
Multiracial	7	5.8	2	9.5	5	5.0	
White	105	86.8	15	71.4	90	90.0	
Socioeconomic status (mean)	48.84	13.58	43.62	13.19	49.94	13.47	.05
Age at depression onset (mean)	12.35	2.04	12.35	2.01	12.35	2.06	.99
Highest level of suicidal ideation (SI) in the past month at study enrollment (C-SSRS)							.20^c^
No SI	9	7.4	1	4.8	8	8.0	
Wish to be dead	16	13.2	5	23.8	11	11.0	
Active SI	36	29.8	10	47.6	26	26.0	
Active SI with methods	16	13.2	1	4.8	15	15.0	
Active SI with some intent	29	24.0	4	19.0	25	25.0	
Active SI with plan	14	11.6	0	0.0	14	14.0	
C-SSRS Lifetime suicide attempt	67	55.4	12	57.1	55	55.0	.89

a C-SSRS: Columbia Suicide Severity Rating Scale [10].

b Groups were collapsed into White and non-White due to some cells having expected counts less than 5.

c Groups were collapsed into low (no SI, wish to be dead), medium (active SI, active SI with methods), and high (active SI with some intent, active SI with plan) due to some cells having expected counts less than 5.

d ECG: electrocardiogram.

Overall feasibility benchmarks were met or exceeded (see Table 2). Participants wore the ECG sensor an average of 14.86 days (SD 7.25) and for 76.21% of the intended minutes (SD 21.01). When worn, valid HRV data was obtained 81.42% of the time (SD 17.33). There were no significant differences in compliance or data quality based on demographic or clinical characteristics (all *P*s≥.14).

Mixed-effects models indicated that compliance and data quality declined over time (compliance: *B*=−0.017, SE=0.002; data quality: *B*=−0.015, SE=0.002; both *P*s<.001) and were higher during sleep than wake (compliance: *B*=0.062, SE=0.007; data quality: *B*=0.094, SE=0.006; both *P*s<.001). There were no significant differences by school attendance (*P*s≥.48).

**Table 2.** Descriptive statistics for ECG compliance and data quality (overall and by sleep/wake and school attendance).

Descriptive statistics	Compliance^a^ (%)		Data quality^b^ (%)	
	Mean	SD	Mean	SD
Overall	76.21	21.01	81.42	17.33
Sleep periods	84.36	36.32	89.46	30.71
Wake periods	77.20	41.96	81.11	39.14
School attendance (% of days)				
Not a school day (48.4%)	80.03	31.57	79.48	27.57
Did not attend school (12.3%)	78.48	32.74	79.60	28.88
Attended school in person (35%)	82.85	29.38	80.64	26.01
Attended school virtually/online (4.4%)	84.28	24.97	91.30	14.64

a Compliance: sensor wear, defined as % of intended minutes the electrocardiogram sensor was worn.

b Data Quality: % of worn minutes with valid heart rate variability data.

## Discussion

Continuous chest-worn ECG monitoring of HF-HRV was feasible in adolescents receiving intensive outpatient treatment for depression and suicidality, producing high data yield across monitoring periods up to three weeks. Feasibility was particularly strong during sleep, consistent with reduced movement and improved electrode contact. Compliance and data quality remained high during school days, suggesting integration of chest-worn monitoring into adolescents’ daily routines is achievable. Declines in feasibility over time likely reflect wear fatigue and chest strap degradation, suggesting an optimal monitoring window of approximately two weeks. Integration with real-time compliance and data quality monitoring (eg, Bluetooth-linked apps) may further improve compliance and data yield.

Ambulatory HF-HRV monitoring via chest-worn ECG is feasible in adolescents at high suicide risk. Potential demographic differences in monitoring participation warrant replication in larger, more diverse adolescent samples. Future studies should also examine feasibility in broader adolescent psychiatric populations and evaluate whether HF-HRV functions as a dynamic marker of short-term suicide risk.

## Abbreviations

ECG: electrocardiogram
HF-HRV: high-frequency heart rate variability

## References

[1] Kivelä L, van der Does WAJ, Riese H, Antypa N (2022). Don’t miss the moment: a systematic review of ecological momentary assessment in suicide research. Front Digit Health.

[2] Stange JP (2024). Integrating dynamic psychophysiological indices across time and contexts: elucidating mechanisms, risk markers, and intervention targets. Psychophysiology.

[3] Beauchaine TP, Thayer JF (2015). Heart rate variability as a transdiagnostic biomarker of psychopathology. Int J Psychophysiol.

[4] Forkmann T, Meessen J, Teismann T, Sütterlin S, Gauggel S, Mainz V (2016). Resting vagal tone is negatively associated with suicide ideation. J Affect Disord.

[5] Tsypes A, James KM, Woody ML, Feurer C, Kudinova AY, Gibb BE (2018). Resting respiratory sinus arrhythmia in suicide attempters. Psychophysiology.

[6] Adolph D, Teismann T, Forkmann T, Wannemüller A, Margraf J (2018). High frequency heart rate variability: Evidence for a transdiagnostic association with suicide ideation. Biol Psychol.

[7] Hamilton JL, Tsypes A, Zelazny J (2023). Sleep influences daily suicidal ideation through affective reactivity to interpersonal events among high-risk adolescents and young adults. J Child Psychol Psychiatry.

[8] Hu X, Sgherza TR, Nothrup JB, Fresco DM, Naragon-Gainey K, Bylsma LM (2024). From lab to life: evaluating the reliability and validity of psychophysiological data from wearable devices in laboratory and ambulatory settings. Behav Res Methods.

[9] Hollingshead AB (1975). Four factor index of social status.

[10] Posner K, Brown GK, Stanley B (2011). The Columbia-Suicide Severity Rating Scale: initial validity and internal consistency findings from three multisite studies with adolescents and adults. Am J Psychiatry.

